# Calcific deposit needling in combination with extracorporeal shock wave therapy (ESWT): A proposed treatment for supraspinatus calcified tendinopathy

**DOI:** 10.1051/sicotj/2018043

**Published:** 2018-10-19

**Authors:** Emilios Pakos, Ioannis Gkiatas, Georgios Rakkas, Dimitrios Papadopoulos, Ioannis Gelalis, Marios Vekris, Anastasios Korompilias

**Affiliations:** 1 Orthopaedic Department, University Hospital of Ioannina, Ioannina Greece; 2 Physiotherapy Department, Salamagka 4 Ioannina Greece

**Keywords:** Supraspinatus, Calcified tendinopathy, Needling, Extracorporeal shock wave therapy

## Abstract

*Background*: Calcified tendinopathy of the rotator cuff is one of the most common conditions concerning the shoulder pathology. It is characterized by a reactive calcification that affects the tendons being part of the rotator cuff. The reported prevalence varies from 2.7% to 22%. Most of the patients can be treated effectively with non-operative measures such as anti-inflammatory drugs, subacromial injection of steroid, physiotherapy, extracorporeal shock wave therapy (ESWT) and needle aspiration irrigation. Results of a treatment combining some of these methods have not been reported.

*Objectives*: The purpose of this study is to present the radiological as well as the clinical results of our proposed protocol which combines drilling of the calcium deposits with xylocaine under ultrasound guidance, with a specific program of physiotherapy for 1 month without the use of NSAIDs.

*Methods*: Sixty-six consecutive patients (68 shoulders) were treated for calcified tendinitis of supraspinatus, which was diagnosed clinically and radiologically, with needle drilling using xylocaine under ultrasound guidance. After the drilling the patient followed a physiotherapy protocol with ESWT which included five visits within a month. After the end of the physiotherapy, the patients were evaluated clinically and radiologically. The Visual Analogue Scale (VAS) for pain and the Disabilities of the Arm, Shoulder, and Hand (DASH) score were measured before and after the end of the therapy.

*Results*: All the patients showed clinical improvement of the symptoms at the follow-up. The mean VAS score showed improvement from 8.1 to 3.3 whereas the mean DASH score was 27 and after the end of the therapy 5. Radiologically all but one calcific deposits were disappeared.

*Conclusions*: The ultrasound-guided drilling of the calcific deposit using xylocaine, in combination with physiotherapy using ESWT provides a reliable alternative treatment for the calcific tendinitis of the supraspinatus

## Introduction

Calcified tendinopathy of the rotator cuff is one of the most common conditions regarding the shoulder pathology. It is characterized by a reactive calcification that affects the tendons that are part of the rotator cuff [1]. The reported prevalence of this condition varies from 2.7% to 22%. Women aged between 30 and 50 years old are the most common affected group [2]. The pathogenesis involves two proposed different processes leading to formation of the calcium deposits in the cuff. The degenerative calcification where the degeneration of the tendon fibers precedes calcification and the reactive calcification where the process of calcification is actively mediated by cells in a viable environment [3].

Concerning the histopathologic findings the calcified tendinopathy consists of three stages: the precalcific, where the tendinous tissue is altered into fibrocartilage, the calcific stage, with a phase of formation and another one of resorption, and the post-calcific stage [4,5]. Due to the fact that the disease is multifocal, with several parts of the tendon undergoing varying stages of the evolutionary process, there are some classification systems trying to personalize the disease's therapy [5].

The management of calcified tendinitis can be divided into two major categories: the conservative treatment and the operative one. The first one includes certain exercises which have to be followed by the patients in combination with non-steroid anti-inflammatory drugs (NSAIDs). The use of NSAIDS is indicated in the acute phase, where the aim is pain relief. Moreover, the extracorporeal shock wave therapy (ESWT) has shown good results and low complication rate [6,7]. It is mostly used in the chronic formative phase with definite radiological evidence of calcium deposits [8]. The needling or puncturing aims to shorten the deposit and accelerate resorption [9]. The operative management is indicated in patients who suffer from the symptoms for 6 months and more and the conservative treatment was not successful. It includes the removal of the lesion either arthroscopically or with open procedures. Lately, ultrasound-guided needle puncture and lavage have been introduced for the therapy of supraspinatus calcified tendinopathy [10,11]. In general, there is no clear evidence of the superiority of a treatment, nevertheless, operative treatment is rarely used.

The purpose of this study is to present a protocol which combines drilling of the calcium deposits with xylocaine under ultrasound guidance, followed by a specific program of physiotherapy including ESWT. In this way, we managed to combine the acute pain relief with the xylocaine, the shortening of the deposit with the needling and the early mobilization with the physiotherapy.

## Materials and methods

From February 2013 to August 2015, 66 consecutive patients (68 shoulders) with a mean age of 54.47 years old (range from 33 to 89 years old) who were first diagnosed with supraspinatus calcified tendinopathy were included in our study. 47 of them were female and 19 were male. In 38 patients the affected shoulder was the right one, in 26 it was the left one, and in 2 patients the problem was bilateral. The mean duration of pain before the patient's visit to our department was 8 months (2 weeks to 2 years). All the demographic data of the patients are shown in Table 1.

**Table 1:** Demographic data of the patients included in the study.

Demographic data of patients	
Number of patients	66
Number of treated shoulders	68
Mean age	54.47 years (33–89)
Men	19
Women	47
Mean duration of pain	8 months (2 weeks–2 years)
Mean follow-up	18 months (6–36)

**Figure 1:**
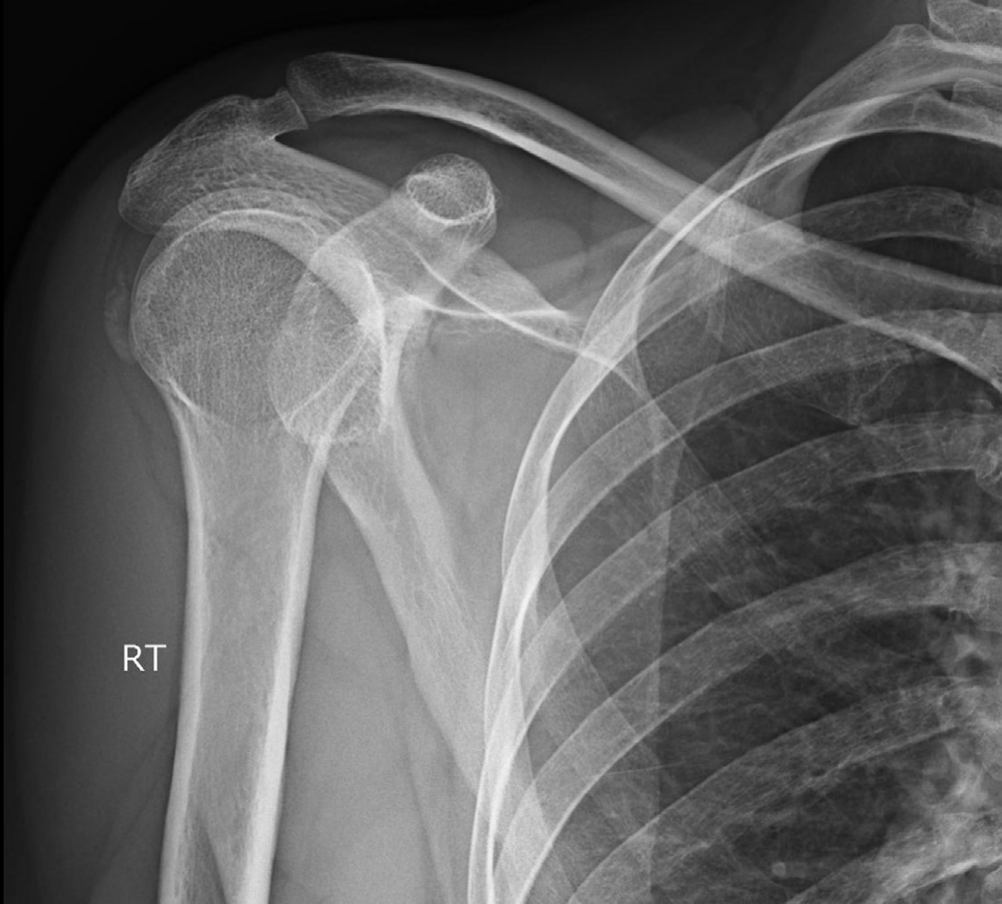
X-ray of a 41-year-old female patient who works as secretary, suffering from calcified tendinopathy before the start of the treatment protocol. The calcific deposit lies on the insertion of supraspinatus.

**Figure 2:**
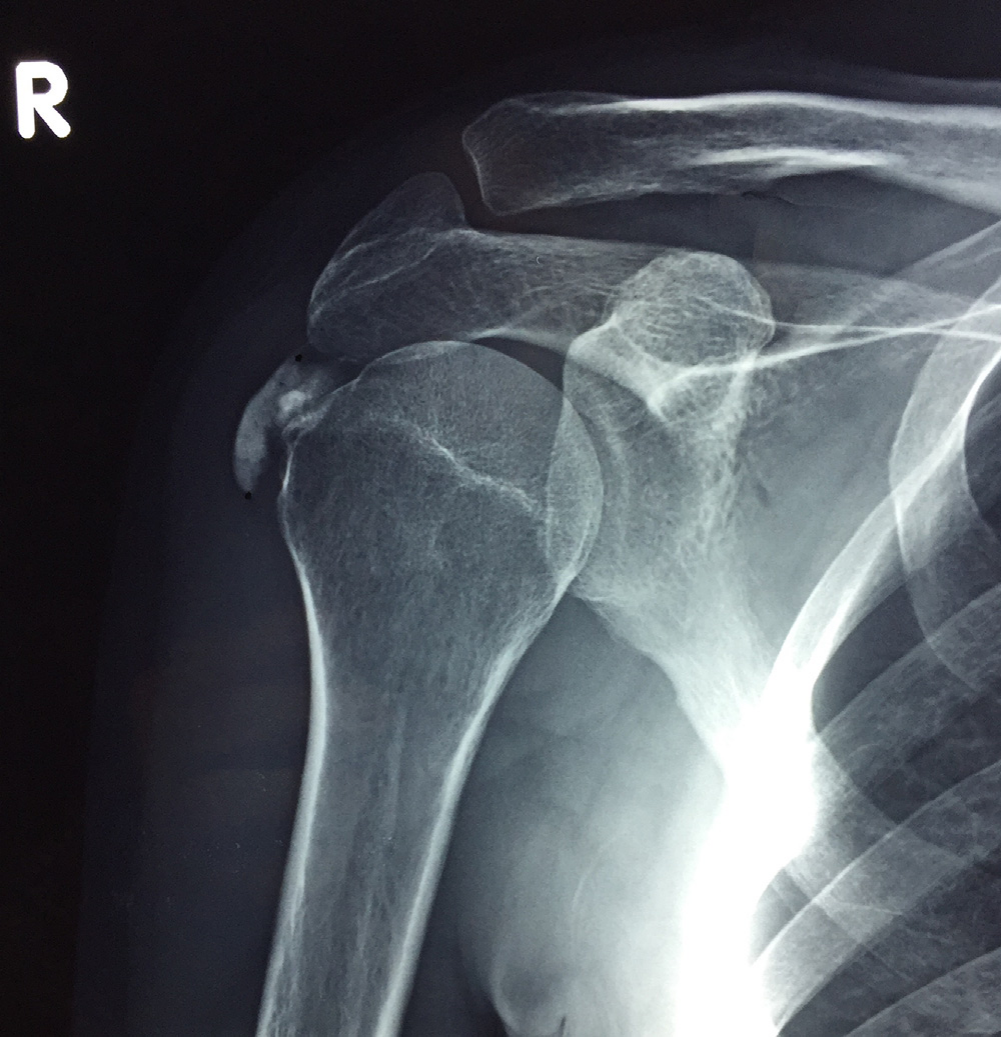
X-ray of a 33-year-old female patient who works as a receptionist. A calcific deposit was found in the insertion of the supraspinatus tendon.

All the patients underwent clinical and radiological examination, which revealed calcium deposits on the supraspinatus tendon (Figures 1 and 2). The calcific deposit was classified radiologically according to Bosworth classification [12]. According to this classification the calcific deposit is characterized as tiny when it is barely visible in fluoroscopy, medium when it is less than 1.5 cm and large when it is more than 1.5 cm.

The patient is placed in sitting position, with the armrest in front of the therapist. After the antiseptic clearance of the shoulder area, under ultrasound guidance a 21G needle with dimension 0.8 × 40 mm was inserted from the lateral side of the shoulder drilling the calcific deposition and injecting xylocaine 2%. The needling of the deposit was performed repeatedly and manually in different sides of the calcific mass without the help of a power drill. After that the needle was removed and the patients were asked to make rotational movements of the shoulder. The total amount of xylocaine that was used in every patient was 5 ml. The arm was kept in brace for 1 week.

After the drilling the patient completed the therapy with 5 visits of physiotherapy using ESWT (Shockmaster 500, Gymna, Uniphy) (every 5–7 days). Since there is no clear consensus about the number of sessions [13,14], the decision about the number of the visits was mostly empirical as in other studies focusing on the effect of ESWT on calcified tendinopathy of the rotator cuff [15,16]. The first visit in the physiotherapist was 3 days after the drilling. The device adjustments were 2.5 bar pressure, 9 Hz frequency and 3000 shocks. The patient was lying on a bed with the affected arm positioned in adduction, the elbow flexed at 90 degrees, and the hand upon the abdomen. The ESWT was applied before the exercises. The probe was placed perpendicular to the calcific deposit and it was applied approximately 1 cm perimetrically of it. No local anaesthesia was applied.

The exercises of the physiotherapy were divided into two main categories: warm up exercises and main exercises. The main exercises of the protocol were divided into 5 different sections: (1) The patient is placed in standing working on the internal and external rotation. (2) The patient in standing position working on the shoulder flexion. (3) The patient is in standing position working on shoulder's abduction. (4) The patient is in standing position, facing a multifunctional bench, holding a bar with both hands close to each other. The patient pulls the bar up, keeping it close to the body, all the way up until it reaches under the chin. The patient tries to keep his elbows as raised as possible and over the level of the bar. The bar is attached via wire and pulleys to a 5 kg weight. (5) The patient is in standing position, slightly forward bended and supporting his healthy hand on the wall. The treated hand is hanging down holding a 2.5 kg weight. The patient performs forward, backward, circular and rotating movements in the treated shoulder joint without bending the elbow. The patient is trying not putt effort in all range of motion (ROM) instead leave the weight to carry on the movement with a small swing at the beginning of the motion. Purpose of the exercise is to apply some traction in the shoulder tendons in each direction and as a relaxing finishing exercise.

After the end of the physiotherapy sessions, the patient underwent new radiological evaluation and then every 6 months. Both Visual Analogue Score (VAS) and the Disabilities of the Arm, Shoulder, and Hand (DASH) score were measured before the start of the therapeutic protocol as well as after the end of the whole therapy. Moreover, the abduction rate was measured before and after the therapy. The mean follow-up of the patients was 18 months (range from 6 to 36 months).

## Results

According to Bosworth classification 15 calcific deposits were characterized as tiny, 33 as medium and 20 as large. All the patients showed significant improvement of the symptoms. There was a significant improvement in the ROM of the shoulder. The mean abduction improved from 81° (rate from 40° to 100°) before the start of the therapy to 110° (range from 90° to 160°). The rate of the improvement was 35.8%. The mean VAS before the start of the therapeutic protocol was 8.1/10 (6.7–9.8), whereas after the end of the therapeutic protocol the mean VAS was 3.3/10 (0–7.2). The percentage of the improvement was 58.02%. Moreover, at the last follow-up of the patients the mean DASH score showed a significant improvement with a decrease from 27(22–32) to 5(2–8). All the results were statistical significant (*p* < 0.001). Additionally, 65 of 66 patients (98.48%) were able to perform their everyday activity with no restriction of the movement of the shoulder. In one patient despite the improvement of the pain, he complained that he was still not able to perform freely his everyday activities.

**Figure 3:**
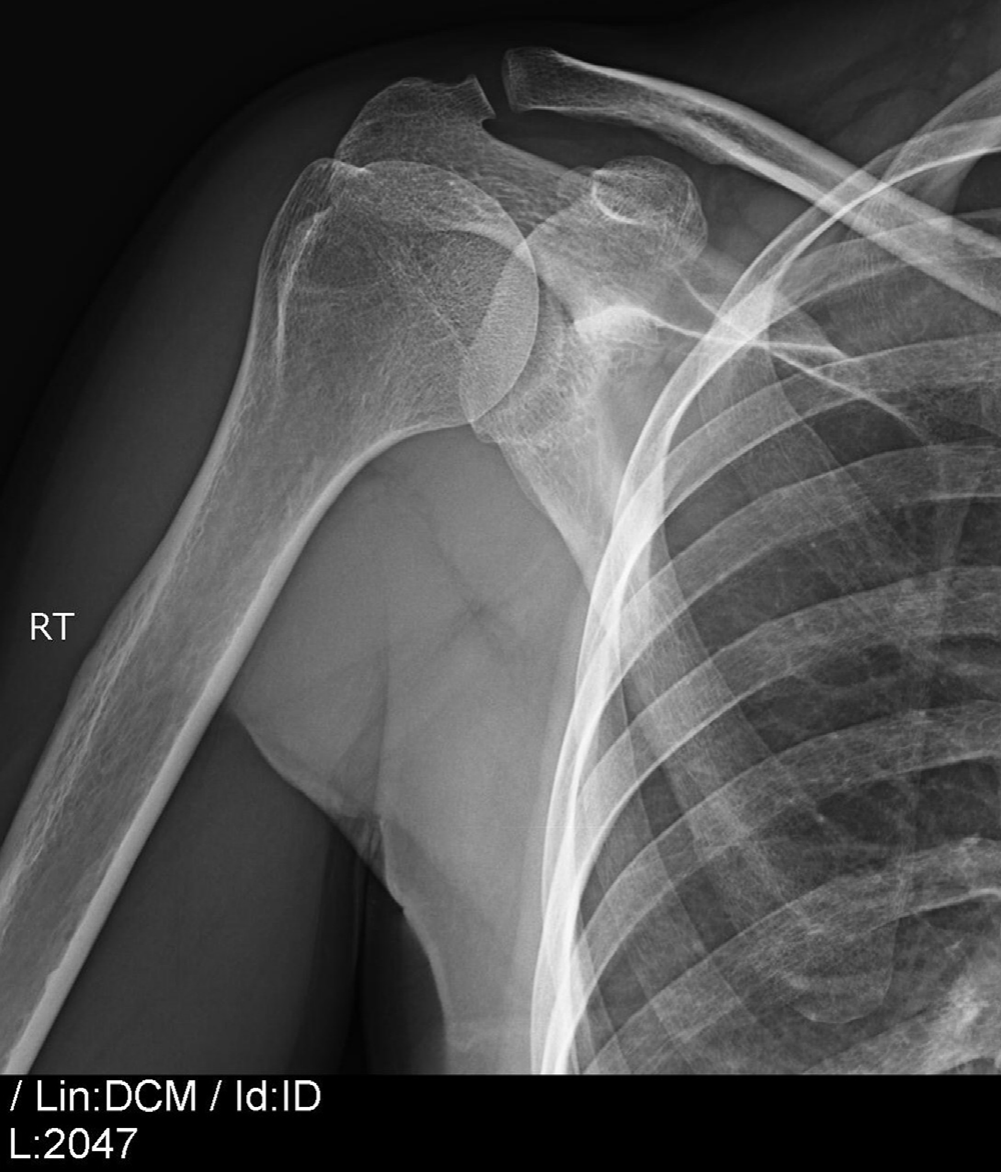
X-ray of the shoulder of the previous patient after the end of the treatment protocol 25 days after the drilling of the deposit.

**Figure 4:**
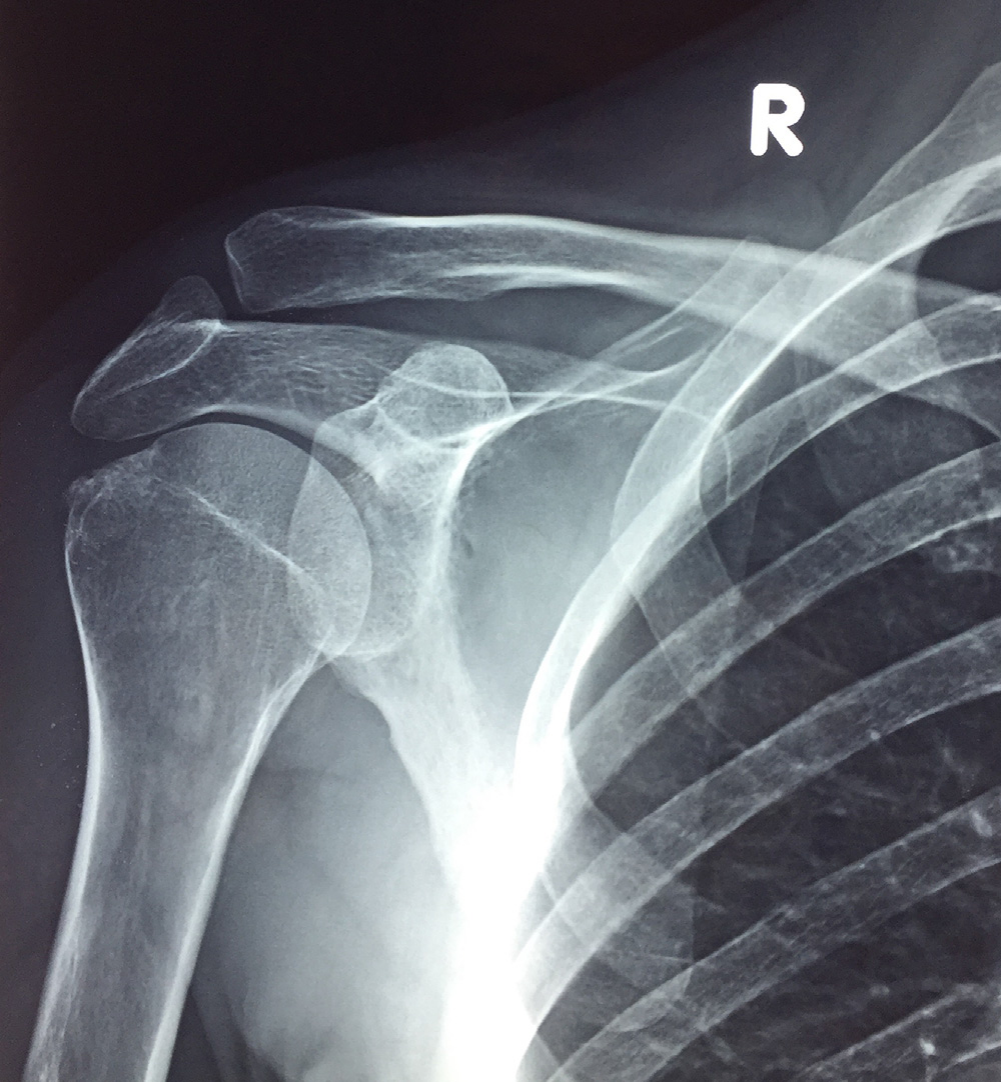
X-ray of the same patient 30 days after the needling and immediately after the end of the whole treating protocol.

As far as it concerns the radiological aspects, in all but one cases the deposit of calcium in the tendon of supraspinatus disappeared in the X-ray that was made after the end the of the five physiotherapy visits (Figures 3 and 4). During the follow-up 65 of the 66 patients did not show signs of deterioration.

## Discussion

The real causes of calcified tendinitis of the rotator till remains a controversial issue. The calcium deposits of the rotator cuff mostly occur in the supraspinatus tendon near its insertion followed by the infraspinatus, teres minor and subscapularis. Calcified tendinitis can also affect more than one tendons at a time [17,18]. Conservative treatment remains the first choice of treatment and most of the times it has successful results [1,19,20].

There are plenty of studies which report good to excellent results after conservative treatment of supraspinatus calcifying tendinitis. Greis et al. [21] report the non-operative treatment options of calcified tendinopathy of the rotator cuff, which include the NSAIDs, the therapeutic ultrasound, the ESWT, the physical therapy and injections of corticosteroid. In a study of Moretti et al. [22] using medium-energy shock wave therapy for calcified tendinopathy of the rotator cuff, 70% of the patients reported satisfactory functional results, whereas in 54% there was disappearance of the calcific deposit in the radiological evaluation.

Minimally invasive techniques such as puncture consist a treatment choice for the physician. The ultrasound-guided barbotage (needling and lavage) has been associated with promising results. Nevertheless, it seems that the corticosteroid injection in the subacromial bursa may have the same impact on the patients in a long-term follow-up [23].

Despite the good reported results of the non-operative treatment of supraspinatus calcified tendinopathy, there is the option of operative treatment. Surgical treatment is reserved for patients in which prolonged conservative therapy has failed and the deposits do not show signs of spontaneous resolution on radiographs [24–26]. Ranalletta et al. [2] reported that 96.2% of the patients, who were operated arthroscopically for the removal of calcific deposit without acromioplasty, were satisfied with the treatment. Later, in 2011 [17], all 56 patients who underwent arthroscopic removal of the calcific deposit were able to return to their level of activity before the beginning of their complaint. Recently Hashiguchi et al. [27] presented a series of patients who were treated with arthroscopic removal and tendon repair for refractory rotator cuff calcific tendinitis. The authors propose that the accurate determination of the deposit even with three-dimensional computed tomography scan is of high importance and the deposit can be removed through a longitudinal incision and end-to-side repair of the cuff. In order to achieve better visualization of the rotator cuff during the operative removal, arthroscopic subacromial bursectomy is proposed. Additionally, in cases of impingement acromioplasty should be performed [28].

Despite the fact that surgical treatment is reserved for patients in which prolonged conservative therapy has failed and the deposits do not show signs of spontaneous resolution on radiographs [24–26], there are controversial issues concerning the arthroscopic treatment of these lesions such as the remove of all calcium deposits and if the remove of the calcific material leaves a hole in the tendon. Moreover, the subacromial decompression is still debated [29–32].

In 2008 Zhu et al. [10] compared the treatment of calcifying tendinitis using ultrasound-guided needle puncture without aspiration of the calcified deposits, with ultrasound-guided needle puncture combined with aspiration of the calcified deposits. They reported good to excellent results in 83% of the patients and they did not find significant statistical difference when compared with the group of patients who were treated with both needle puncture and aspiration. Later, in 2014 Castillo-Gonzalez et al. [11] reported that in 83.78% of the patients treated with ultrasound-guided percutaneous needle lavage the calcific deposits were disappeared after 1 year whereas 89.26% of the patients were pain free. All the patients after 2 years follow-up had a decrease in pain as well as in the size of the calcification.

In a retrospective comparative study between arthroscopic surgery and shock wave therapy for chronic calcifying tendinitis [33], the authors conclude that shock wave therapy is equivalent to arthroscopy, and so shock wave therapy should be preferred due to its noninvasiveness. In addition, the authors support that ESWT should be performed when adequate conservative approach has failed.

Despite the different proposed protocols for the treatment of calcified tendinopathy in the international literature, the information concerning the epidemiology and the prognostic factors is rare. In 2016, de Witte et al. [34] collected the demographic characteristics and treatment of 342 patients. According to the authors the female gender, the dominant arm the bilateral disease, the longer duration pf the symptoms and the presence of multiple calcified deposits are factors which predispose for inferior outcome.

Our study presents a treatment protocol which includes the drilling of the calcific deposit and ESWT with very promising results. It is the first described technique which takes advantage of both needle puncture as well as of the well-established beneficial effect of the ESWT. In our series of patients, approximately 98% were satisfied with the results and there was a movement improvement approximately 58%.

Despite the promising results, which are similar with the results of the current international bibliography, the current study has several limitations. At first, a bigger number of patients is needed in order to be able to extract secure results about the proposed protocol treatment as well as a longer follow-up period for every patient. Moreover, the present study consists a retrospective study, and there is absence of control group as a comparative group in order to assess better the results of the presented method.

In conclusion, conservative treatment should be the first choice in the treatment of the patients who suffer from calcified tendinitis of the supraspinatus with the use of ESWT and operative treatment for the removal of the calcific deposit should be considered after the failure of the conservative treatment. Based on our study, it seems that a combination of the use of ESWT with the drilling of the deposit using a 21G needle as well as the xylocaine injection offers a very promising therapeutic protocol for these patients and especially for those who favor the non-operative treatment.

## Conflict of interest

The authors declare no conflict of interest.
